# Comentários Sobre o Eletrocardiograma do Atleta nas Diretrizes da Sociedade Brasileira de Cardiologia sobre a Análise e Emissão de Laudos Eletrocardiográficos – 2022

**DOI:** 10.36660/abc.20220670

**Published:** 2022-12-20

**Authors:** Filipe Ferrari, Anderson Donelli da Silveira, Ricardo Stein

**Affiliations:** 1 Programa de Pós-Graduação em Cardiologia e Ciência Cardiovasculares Universidade Federal do Rio Grande do Sul Porto Alegre RS Brasil Programa de Pós-Graduação em Cardiologia e Ciência Cardiovasculares – Universidade Federal do Rio Grande do Sul, Porto Alegre, RS – Brasil; 2 Grupo de Pesquisa em Cardiologia do Exercício Universidade Federal do Rio Grande do Sul Porto Alegre RS Brasil Grupo de Pesquisa em Cardiologia do Exercício (CardioEx) – Universidade Federal do Rio Grande do Sul, Porto Alegre, RS – Brasil; 3 Hospital de Clínicas de Porto Alegre Porto Alegre RS Brasil Hospital de Clínicas de Porto Alegre, Porto Alegre, RS – Brasil; 4 Universidade Federal do Rio Grande do Sul Porto Alegre RS Brasil Universidade Federal do Rio Grande do Sul, Porto Alegre, RS – Brasil

**Keywords:** Eletrocardiografia, Atletas, Guia de Prática Clínica

Prezado Editor,

Foi com grande interesse que tivemos acesso ao documento intitulado “Diretrizes da Sociedade Brasileira de Cardiologia sobre a Análise e Emissão de Laudos Eletrocardiográficos – 2022”, publicado na Edição de setembro dos Arquivos Brasileiros de Cardiologia (ABC),^1^ elaborado por um grupo de especialistas brasileiros no assunto. É nossa opinião que uma atualização se fazia necessária, tendo sido a mesma detalhada e de agradável leitura.

No entanto, chamamos atenção para duas informações apresentadas sobre a interpretação do eletrocardiograma do atleta que nos trouxeram preocupação. A primeira se encontra na seção “Sobrecargas das Câmaras Cardíacas” (Página 18), na qual os autores descrevem que o Critério de Sokolow-Lyon NÃO DEVE ser utilizado para avaliação da sobrecarga ventricular esquerda (SVE) EM ATLETAS.

Discordamos dessa afirmativa e nos baseamos para tanto no *International criteria for electrocardiographic interpretation in athletes: Consensus statement* ,^2 - 4^ do qual o autor sênior desta carta foi um dos autores. Nesse documento, publicado simultaneamente em três periódicos de alto impacto,^2 - 4^ o Critério de Sokolow-Lyon (soma da onda S em V1 + onda R em V5 ou V6 >3,5 mV) serve sim para definir SVE nesse grupo de indivíduos treinados (Tabela 1 do documento).^2^ É fato que embora existam vários critérios para definir a SVE em atletas, o de Sokolow-Lyon ainda é o mais utilizado. Cabe salientar que uma série de estudos que avaliaram o eletrocardiograma do atleta também utilizaram esse critério para avaliar tal sobrecarga.^5 - 12^

Neste ponto, nos permitimos fazer uma suposição: Talvez os autores da “Diretrizes da Sociedade Brasileira de Cardiologia sobre a Análise e Emissão de Laudos Eletrocardiográficos – 2022”^1^ quiseram dizer que o critério de Sokolow-Lyon não deve ser usado isoladamente para definir SVE em atletas, visto que estas alterações são consideradas variantes da normalidade nesse cenário? Contudo, ao escrever “não deve ser utilizado em atletas”, nos parece que tal assertiva pode causar confusão ao leitor, trazendo uma informação incorreta.

A segunda preocupação que veio à tona ao lermos o manuscrito diz respeito à seção 12.1.2 (Página 29), na qual os autores das Diretrizes colocam um complexo QRS com duração ≥160 ms como anormal. De fato, eles têm razão. No entanto e em contraste, os critérios internacionais para interpretação do eletrocardiograma do atleta^2^ destacam uma duração do QRS como sendo anormal já quando ≥140 ms (Figura 1 do documento),^2^ e não somente ao alcançar a duração ≥160 ms.

Por fim, é importante destacar que os mesmos autores das recentes Diretrizes Brasileiras de Eletrocardiograma^1^ fazem referência aos critérios internacionais para interpretação do eletrocardiograma do atleta^2^ em seu documento. Dessa forma, acreditamos que essas correções pontuais por nós propostas possam ser consideradas, sem que isso desmereça em nada o importante e certamente árduo trabalho realizado na confecção dessa Diretriz.
