# Supplementary material for: Comentários Sobre o Eletrocardiograma do Atleta nas Diretrizes da Sociedade Brasileira de Cardiologia sobre a Análise e Emissão de Laudos Eletrocardiográficos – 2022
Source: Arq Bras Cardiol. 2022 Dec 20;120(1):e20220670. [Article in Portuguese] doi: 10.36660/abc.20220670 (PMC9833214; doi:10.36660/abc.20220670)
Supplement: Translation

# Translation: Comments Regarding the Athlete’s Electrocardiogram in the Brazilian Society of Cardiology Guidelines on the Analysis and Issuance of Electrocardiographic Reports – 2022

**Authors:** Filipe Ferrari, Anderson Donelli da Silveira, Ricardo Stein

**Affiliations:** 1 Brazil Programa de Pós-Graduação em Cardiologia e Ciência Cardiovasculares – Universidade Federal do Rio Grande do Sul, Porto Alegre, RS – Brazil; 2 Brazil Grupo de Pesquisa em Cardiologia do Exercício (CardioEx) – Universidade Federal do Rio Grande do Sul, Porto Alegre, RS – Brazil; 3 Brazil Hospital de Clínicas de Porto Alegre, Porto Alegre, RS – Brazil; 4 Brazil Universidade Federal do Rio Grande do Sul, Porto Alegre, RS – Brazil

**Keywords:** Electrocardiography, Athletes, Practice Guideline

## Dear Editor,

It was with great interest that we had access to the document entitled *“Brazilian Society of Cardiology Guidelines on the Analysis and Issuance of Electrocardiographic Reports – 2022* ”,^1^developed by Brazilian experts on the subject. It is our opinion that an update was necessary, as it is detailed and pleasant to read.

However, we draw attention to two pieces of information presented about the interpretation of the athlete’s electrocardiogram that caused us concern. The first is found in the section “ Cardiac Chambers Hypertrophy” (Page 18), in which the authors describe that the Sokolow-Lyon Criteria SHOULD NOT be used to determine left ventricular hypertrophy (LVH) IN ATHLETES.

We disagree with this statement and based it on the *International criteria for electrocardiographic interpretation in athletes: Consensus statement* ,^2 - 4^of which the senior author of this letter was one of the authors. In this document, published simultaneously in three journals with high impact factor,^2 - 4^the Sokolow-Lyon Criteria (S wave in V1 + R wave in V5 or V6 >3.5 mV) does serve to define LVH in this group of trained individuals (Table 1 of the document).^2^It is a fact that although there are several criteria to define LVH in athletes, Sokolow-Lyon is still the most used. It should be noted that a series of studies that evaluated the electrocardiogram in athletes also used this criterion to assess such hypertrophy.^5 - 12^

At this point, we allow ourselves to make an assumption: could it be that the authors of the *“Brazilian Society of Cardiology Guidelines on the Analysis and Issuance of Electrocardiographic Reports – 2022* ”,^1^meant that the Sokolow-Lyon criteria should not be used alone to define LVH in athletes, since these alterations are considered normal variants in this scenario? However, when writing “should not be used in athletes”, it seems to us that such an assertion could cause confusion to the reader, bringing incorrect information.

The second concern that came to the fore when reading the manuscript relates to section 12.1.2 (Page 29), where the Guidelines authors place a QRS complex duration ≥160 ms as abnormal. Indeed, they are right. However, in contrast, the *International criteria for electrocardiographic interpretation in athletes* ^2^highlight a QRS duration as being abnormal even when ≥140 ms (Figure 1 of the document),^2^and not only when reaching a duration ≥160 ms.

Finally, it is important to highlight that the same authors of the recent Brazilian Guidelines of Electrocardiographic^1^make reference to *the International criteria for electrocardiographic interpretation in athletes* ^2^in their document. Thus, we believe that these specific corrections proposed by us can be considered, without this in any way detracting from the important and certainly hard work carried out in the preparation of these Guidelines.
