# Supplementary material for: Comentários Sobre o Eletrocardiograma do Atleta nas Diretrizes da Sociedade Brasileira de Cardiologia sobre a Análise e Emissão de Laudos Eletrocardiográficos – 2022
Source: Arq Bras Cardiol. 2022 Dec 20;120(1):e20220670. [Article in Portuguese] doi: 10.36660/abc.20220670 (PMC9833214; doi:10.36660/abc.20220670)
Supplement: Related texts

# Reply: Carta-resposta

**Authors:** Nelson Samesima

**Affiliations:** 1 Instituto do Coração Hospital das Clínicas da Faculdade de Medicina da Universidade de São Paulo São Paulo SP Brasil Instituto do Coração (InCor) do Hospital das Clínicas da Faculdade de Medicina da Universidade de São Paulo (HCFMUSP), São Paulo, SP – Brasil

Agradeço o envio dos apontamentos feitos pelo(s) colega(s) referentes à nova Diretriz sobre Análise e Emissão de Laudos Eletrocadiográficos, a qual será, em breve, lançada pelos Arquivos Brasileiros de Cardiologia.

1. As duas observações feitas sobre a Diretriz são absolutamente pertinentes. Apesar de ter sido extensamente revisada (versões em Português e em Inglês), alguns erros podem ter passado despercebidos.
2. Sim, faltou a palavra ISOLADAMENTE na página 16 (Item 6.1.4.2 Índice de Sokolow Lyon), sendo a frase correta: “Isoladamente, não deve ser utilizado em atletas.”

Houve um erro de digitação na página 27 (Item 12.1.2 Achados Eletrocardiográficos Atuais (Grupo 2)), sendo o correto: “Duração do QRS^3^140ms.”

Mais uma vez, agradeço aos colegas.

# Reply

**Authors:** Nelson Samesima

**Affiliations:** 1 Brazil Instituto do Coração (InCor) do Hospital das Clínicas da Faculdade de Medicina da Universidade de São Paulo (HCFMUSP), São Paulo, SP – Brazil

Thanks for sending the notes made by the colleague(s) regarding the new Directive on Analysis and Issuance of Electrocardiographic Reports, which the Brazilian Archives of Cardiology will soon release.

1. The two observations made about the Guideline are absolutely pertinent. Despite having been extensively revised (Portuguese and English versions), some errors may have gone unnoticed.
2. Yes, the word ISOLATEDLY was missing on page 16 (Item 6.1.4.2 Sokolow Lyon Index), the correct sentence being: “Isolatedly, it should not be used in athletes.”

There was a typing error on page 27 (Item 12.1.2 Current Electrocardiographic Findings (Group 2)), which is correct: “QRS duration^3^140ms.”

Once again, thanks to colleagues.
